# Imagery: Interference, facilitation, and theory

**DOI:** 10.3758/s13414-026-03251-6

**Published:** 2026-04-18

**Authors:** Adam Reeves

**Affiliations:** Department of Psychology, Northeastern University, Boston, MA 02115 USA

**Keywords:** Imagery, Signal detection theory

## Abstract

Visual images in the vicinity of visual targets interfere with target discrimination (*d′*) at high levels of performance but facilitate discrimination at low levels. Here, I review the background literature that supports this conclusion and theorize that an image showing features matching those of the target boost sensitivity at every level of performance, but images of all types (including matching ones) also add multiplicative noise. This noise increases in proportion to the signal level, eventually overcoming the boosting effect and causing interference (i.e., the Perky effect). Experiment 1 demonstrates that image type (vivid or weak, static or dynamic) has no effect on the changeover from facilitation to interference; the critical factor is signal level. Equations for both the mean and standard deviation of *d′* are derived and shown to fit data from Experiment 1, two previous large-scale studies from my lab, and a recent study from another lab.

## Introduction

Perception and mental imagery are clearly distinct, yet images and percepts interact, in that imagining common sounds makes it harder to hear concurrent sounds (Ellson, 1942) and imagining pictures makes it harder to see (Perky, 1910). Interaction is consistent with fMRI scans showing that the cortical areas responsive to imagery and perception overlap considerably (Dijkstra et al., 2019; Ganis, et al., 2004; Pearson, 2019). This paper reviews and discusses interactions between perception and imagery, concentrating on the visual modality where the bulk of the research has been done.

By ‘imagery’ I refer to *voluntary mental visual images* of well-known, concrete visual stimuli, such as a mental picture of a dog, or a well-known shape or pattern, such as a letter or grating. Afterimages are involuntary and thus excluded. Abstract images, say of justice, may also interact with perception, but this is unknown, so these are not discussed. Concrete voluntary images can both facilitate and interfere with vision, and my purpose in this paper is to explain why this is the case, using the theory of signal detection (TSD). The model, as formulated, predicts that the level of performance, rather than the type of image, controls whether imagery helps or hinders perception, and this prediction is tested in Experiment 1. First, I give a brief historical review of the ‘Perky effect’ and a summary of TSD.

### Perky effect

The visual interaction has been analyzed extensively since C. W. Perky (1910) showed that visual images raise the threshold for visual perception, in part because her subjects confused images with percepts (Segal & Fusella, 1970). Although nonconfusable images can also raise thresholds (Craver-Lemley & Reeves, 1987), still, Perky’s study has been of philosophical interest since it bears on the criterion for distinguishing reality from imagination (e.g., Todd, 2024). David Hume (1739/1992) had claimed that vivid appearances are experienced as real and less vivid ones as imaginary, as he sought to put the reality/imaginary distinction on an empirical and phenomenalistic basis. According to Hume’s pupil Thomas Reid, Hume’s idea requires that ‘sensation, memory, belief, and imagination, when they have the same object, are only different degrees of strength and liveliness in the idea’, which he found nonsensical (Reid, 1764/1977, p. 15). Indeed, vividness cannot be the only criterion; fidelity, for example, must also matter, since it is often possible to imagine vivid but impossible objects (Sartre, 1940), and images are sometimes controllable, in that they can be summoned up and moved around, whereas reality is not. I therefore use the more abstract term ‘strength’ to refer to some combination of vividness, plausibility, fidelity, controllability, and any other property that can help distinguish imagery from reality. This definition of strength goes beyond Hume, who assumed only that vividness was phenomenally accessible, but is in line with the ideas of Reid (1764/1977; see Reeves & Dresp-Langley, 2017).

A revised version of Hume’s idea, then, is that a ‘reality’ decision is the more likely, the stronger (the more vivid, more plausible, and less controllable) the phenomenal experience. Conversely, an ‘imaginary’ decision is more likely if the phenomena of vision are weaker—less vivid, less realistic, and more controllable. To the extent that the features defining strength are conscious (i.e., ‘phenomenal’) and that the imagery versus reality decision depends on strength, weaker phenomena will be typically categorized correctly as imaginary and stronger ones as real. Only when the image is relatively strong will it be (erroneously) confused with reality. As Dijkstra and Fleming (2023) point out, ‘a consequence of this account is that when virtual or imagined signals are strong enough, they become subjectively indistinguishable from reality’. This paper will model this idea, although I note here individuals reporting very vivid imagery have slightly smaller Perky effects, not larger ones, than those with moderate imagery (Craver-Lemley & Reeves, 1987).

### The theory of signal detection (TSD)

Following Segal and Fusella (1970), the Perky effect can be formalized in the theory of signal detection (TSD; D. M. Green & Swets, 1966). The term ‘signal’, S, refers to neural (visual or auditory) signals evoked by an external stimulus. The signal has a quantitative value related by experiment to some physical measure of the stimulus and its environment such as amplitude, energy, contrast, or duration. In TSD detectability, *d′*, increases with S/N, the signal-to-noise ratio, so if the noise is fixed and S increases, *d′* must also increase (as in Eq. 1, below). If the noise also increases with S, then a constraint can be imposed to ensure that d′ still increases with S, as in Eq. 2, below.

Since an energetic stimulus may be made hard to see by noise, or made hard to discriminate from another such stimulus, it is often best to think of the physically measured stimulus level (SL) not in absolute terms but in terms of contrast against a measured background or other stimuli. The neural signal, S, is then assumed to be an increasing monotonic function, f, of stimulus level, SL, such that S = f(SL), where S = f(0) = 0, and, if a sensory threshold, T, exists, S = 0 if SL < T (see Reeves, 2023). Ideally both the function f(SL) and the appropriate physical measure of SL are known prior to the introduction of imagery. Note that some physical measures (e.g., amplitude; contrast) may be negative as well as positive, in which case the absolute value or square is taken to define SL in the function f. In many cases f(SL) is known to obey a theoretical law, such as the power law or Weber’s law, in the absence of imagery. Given that few imagery studies have varied SL, the default assumption (as made explicit in Eq. 1, below) is that the transform, f, from stimulus level, SL, to neural signal, S, is the same whether an image is present or not. Any effect of the image will be due to a later, top-down modulation of S, a reasonable assumption but one that needs empirical verification.

In the foundational experiments of Sydney Segal, half the trials contained a stimulus, and half did not, at random. Her subjects either imagined a series of objects, one on each trial, in a block of ‘imagery’ (IM) trials, or avoided having any imagery in separate blocks of ‘No image’ (NI) or, in Segal’s terms, ‘discrimination’ trials. Imagery lowered sensitivity (*d′*) relative to NI trials in every study she ran (Segal & Fusella, 1969, 1970; Segal & Gordon, 1969; Segal & Nathan, 1964). She introduced the term ‘Perky effect’ to describe the imagery-induced sensitivity loss, as imagery hardly affected the response criterion, only *d′*. However, since only one fixed stimulus level was employed per experiment, the relation between d′ and SL was not specified, only that d′ was lower for the same stimulus in IM than in NI.

Segal described imagery-perceptual interactions using TSD in part because the Yes/No experiment with confidence ratings operationalizes the classical terms in the philosophy of perception (Segal, Personal communication, 1970). Following Reid (1764/1977), the term ‘image’ is an appearance of something known not be present, whereas a ‘percept’ refers to a valid appearance of something real (a hit): thus, I ‘perceive a dagger before me’ only if I see a dagger *and* it is there *and* I am confident of it (see Reeves & Dresp-Langley, 2017). If it is not there, it is a ‘false alarm’, a confident false alarm being an hallucination (i.e., a certainty that a stimulus is present when it is not.) A confident correct rejection implies a certainty of absence. These philosophical terms exclude pure guesses based on zero information, as does TSD, but guesses may be made rare if the experimental stimuli are chosen appropriately, and may be ignored in the calculation of sensitivity except at very high levels of *d′* (Kontsevich et al., 2002; Reeves, 2023). Thus, the use of TSD was attractive to Segal and has been to us and to others (e.g., Ishai & Sagi, 1995, 1997a, 1997b).

I note here that some ‘false alarms’ may be true perceptions of momentary events in the noise that fortuitously resemble the target. Studies of this phenomenon using, say, the ‘twin noise’ method (Beard & Ahumada, 1999) have not been made in the imagery literature, and so here, all ‘false alarms’ are taken to be misclassifications of the noise by the subject rather than the experimenter. I also note that the two-alternative forced-choice (2AFC) method has frequently been used to study the Perky effect, as being more efficient than the Yes/No method, but 2AFC sacrifices the assessment of confidence. However, in a direct comparison, Reeves (1981) found that the average reduction in *d′* in Imagery (IM) compared with No Imagery (NI) was the same with 2AFC as with Yes/No methods.

Reid’s idea that perception is sensation followed by a correct decision is also illustrated by TSD, in which a sensory ‘detector’ is followed by a ‘decider’ whose operation is independent of the detector. In theory, the ‘decider’ is a cortical network whose input is sensory and whose output is a detection which may, if labelled, leads to recognition. To illustrate, a pattern of line segments may be sensed, and the decision is formed that a letter *A* has been presented; together, these two steps imply that an *A* has been detected, matched to a known letter, and so recognized. Since input signals will evoke responses in many different units, further processing may be required to find the best match (e.g., Grossberg, 2017). Processing errors can arise at either level in TSD. The signal may be so corrupted by noise that the wrong information is delivered to the decider, or the signal may be correctly encoded but the decider fails to determine the best match. Since decision errors increase with the number of potential competitors, decision noise can be estimated empirically, but this remains to be done in imagery research.

### Internal and external noise

The major contribution of TSD to sensory psychophysics was to think of the signal (S) in terms of the noise (N); only signals strong enough to overcome the noise will be detectable (i.e., visible or audible). Noise stems from both internal and external sources, and these are assumed to be distributed across trials as independent random variables (usually Normal) with variances σe^2^ and σi^2^. Any decision noise σd, and any within-trial variance σw, is presumed to be uncorrelated with σe and σi. Thus the total variance in N is σn^2^ = σe^2^ + σi^2^ + σw^2^ + σd^2^. Within-trial noise may be assessed physiologically or by psychophysical ‘multiple looks’ methods, but this has not been done in the Perky literature, and decision noise has also not been tested. When the noise sources are unknown and lumped together, TSD takes the total noise to be normal with mean 0 and variance 1, so that σn = 1.0.

### Perky effect: Detection or decision

Segal and Fusella (1970) first asked whether visual and auditory thresholds were raised by imagery at the sensory (detection) or decisional stage. A stimulus was presented on half the trials. Their subjects rated how certain they were of each Yes (‘stimulus present’) or No (‘no stimulus’) response. Such ratings trace out a ‘receiver operating curve’ or ROC, when the hit rate, P(hit), is plotted against the false-alarm rate, P(fa), on normal–normal axes, at each level of certainty. Segal and Fusella (1970) obtained P(hit) and P(fa) from a block of imagery (IM) trials, and separately, P(hit) and P(fa) from a block of no imagery (NI) trials. They found that imagery lowered the ROC, indicating that sensitivity (*d′*) was reduced, but had no apparent effect on the decision stage. They reported both *d′* and the likelihood ratio at the criterion. The likelihood ratios averaged 3.96 in NI (No Imagery) and 3.71 in IM (Imagery; Segal & Fusella, 1970, Table 2), which did not differ statistically, so they concluded that imagery lowered sensitivity but did not affect the response criterion.

**Fig. 1:**
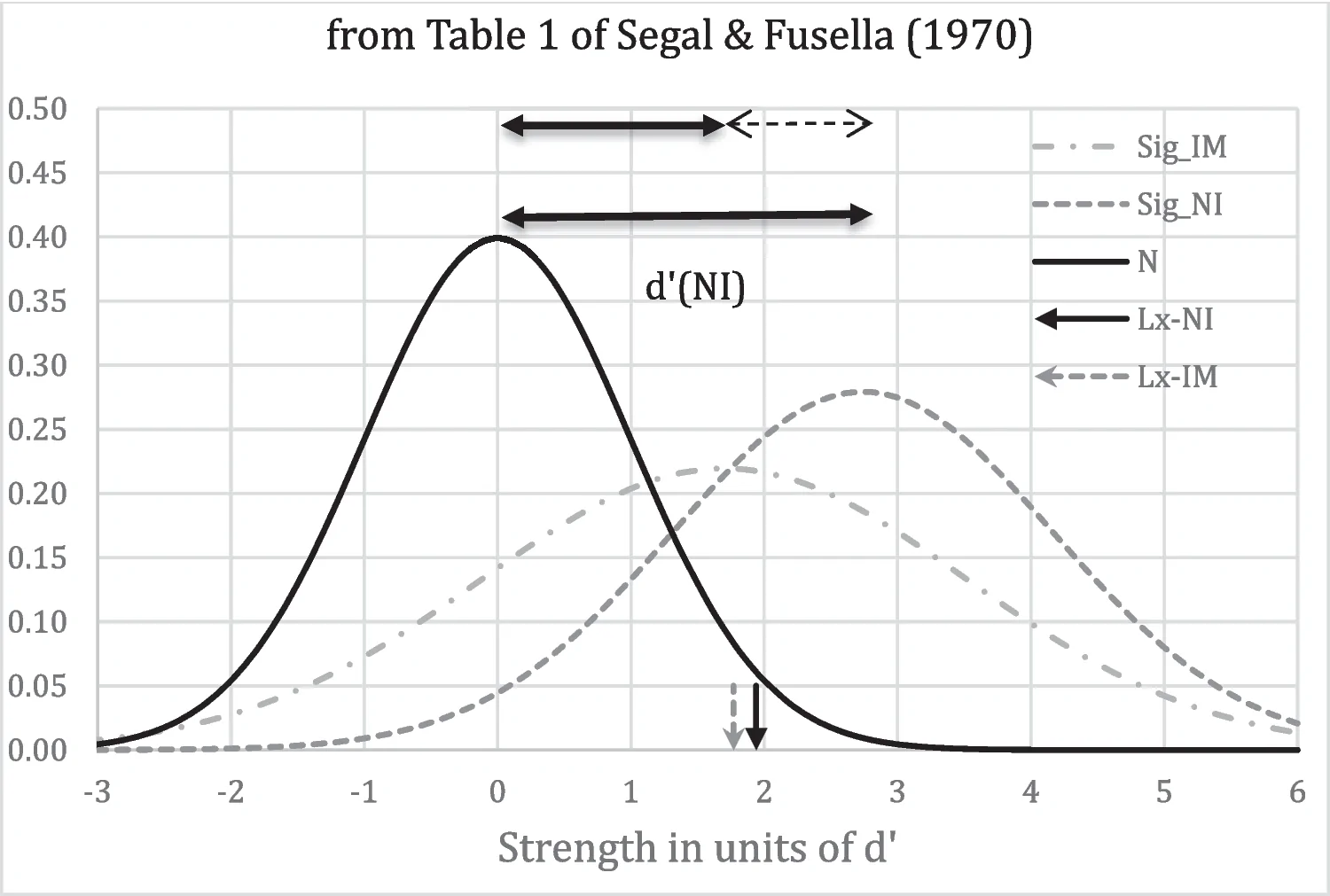
The effect of visual imagery on vision (Segal & Fusella, 1970, Experiment 2). Curves plot hypothetical distributions of strengths, taken across trials with noise alone (solid curve, N) or with a signal and visual imagery (dot-dash curve, Sig_IM) or a signal and No Imagery (dashed curve, Sig_NI). Vertical arrows show the criteria, the − Z(Pfa) scores, 1.94 in NI and 1.78 in IM, corresponding to the reported likelihood ratios, Lx, 3.96 in NI and 2.63 in IM. Signals whose strengths are to the right of the vertical arrows are reported as stimuli (i.e., hits in Sig trials and false alarms in No trials), and those to the left as no stimuli (i.e., misses in Sig trials and correct rejections in N [noise alone] trials [solid curve]). The reported *d′* in Sig_NI was 2.74, and that in Sig_IM was 1.70. The difference, *d′* = 1.04, is the reduction in *d′* due to imagery (i.e., the Perky effect). The Sig_NI and Sig_IM curves are 1.4 and 1.8 times wider than the N curve, to match the inverses of their ROC slopes

A Yes/No TSD scheme for imagery is shown in Fig. 1, based on Experiment 1 of Segal and Fusella (1970; their Fig. 1 and Table 2). The abscissa represents fluctuations in the strength of the signal, low on the left and high on the right. Each abscissa represents the mean strength on a trial in one of the four conditions. The probability density curves represent fluctuations in strengths *across* trials, not within trials, so each trial is quantified by a single strength. The solid curve represents N or noise alone, the distribution of strengths on stimulus-absent trials. In TSD, noise is always present, and the signal adds to the noise, so signal trials are S + N. The two dotted curves represent strengths on stimulus-present trials with imagery (IM) and without imagery (NI). Both curves are positioned to the right of the N curve, as they include N, but the curve with imagery, Sig_IM, is placed leftward of that with no imagery, Sig_NI, to illustrate the Perky effect found by Segal and Fusella (1970).

In Fig. 1, both image and stimulus were visual. The mean *d′* in no imagery, *d′*(NI), was 2.74. That in imagery, *d′*(IM), dropped to 1.70. The Perky effect equals the difference, 1.04 *d′* units (Segal & Fusella, 1970; Table 2).

The criterion for deciding ‘Yes’ is shown by the black vertical arrows in Fig. 1, which are the *z*-axis values corresponding to the likelihood ratios reported by Segal and Fusella (1970). The probability P(hit) of reporting the stimulus when present is represented by the area under the S + N distribution to the right of the arrows. The false alarm rate, P(fa), the chance of reporting Yes given only noise, by the area under the N curve to the right of the arrows. The criteria, − z(Pfa) = 1.94 in NI and 1.77 in IM, control the response—if the total strength is greater than this, the observer reports Yes. Note that there is only one curve for stimulus-absent (i.e., noise-alone) trials, not two, in Fig. 1, because, as already stated, the default total noise in TSD is Normal with zero mean and unit variance (sn = 1). This is not essential, and indeed, Eq. 2, below, fit data with more noise in IM than in NI.

### d’ and c with noisy signals

Segal and Fusella (1970, Fig. 1) plotted the ‘receiver operating characteristic ‘ (ROC) curve found by placing *z*(Phit) and *z*(Pfa) on normal-normal axes for each level of confidence, where *z*(.) refers to the *z*-score. If the signal is constant across trials, the signal plus noise distribution, S + N, has the same variance as N, so σs = σn, and the slope, s, of the ROC curve will be 1.0. If S fluctuates across trials, with variance σsg^2^, then the S + N distribution is wider with variance σs^2^ = σsg^2^ + σn^2^, and the ROC slope is s = σn^/^σs = 1/σs, given unit variance noise, σn = 1. ROC data permit assessment of the slope, s. By definition, *d′* = (2/(1 + s)) [*z*(Phit) − szP(fa)] which simplifies to the usual *d′* = z(Phit) − z(Pfa) if s = 1. The bias is c = − (2 s/(1 + s)^2^[z(Phit) + z(Pfa)], which simplifies to c = − (1/2) [z(Phit) + z(Pfa)] if s = 1 (MacMillan & Creelman, 1991, pp. 71–74).

The concept of a ‘slope’ (s) requires that a plot of z(Phit) versus s(Pfa) is linear. The ROCs shown in Fig. 1 of Segal and Fusella (1970) were strongly curved for P(fa)’s between 0.005 and 0.020, but were quite linear for higher Pfa’s. I estimated slopes of s = 0.70 in NI and s = 0.55 in IM from their Fig. 1 for the range 0.02 < Pfa < 0.55. Slope-adjusted, *d′*(NI) = 2.54 and *d′*(IM) = 1.16, for a slightly larger Perky effect of 1.38 *d′* units. The slope-adjusted biases, c = + 0.55 in NI and + 0.85 in IM, are conservative compared to no bias (c = 0), but similar—the subjects required almost equally strong signals to report ‘Yes’ in IM and NI. Such similarity is meaningful as IM and NI trials were blocked; when randomized, Pfa is common to both conditions.

### Perky effect: Explanations

Various explanations have been offered for the Perky effect, as reviewed in Craver-Lemley and Reeves (1992), and summarized here.

#### Optics

It was speculated that having an image might decrease sensitivity by altering the optics of the eye, namely, gaze direction, pupil diameter, or optical focus (Finke, 1985; Peterson & Graham, 1974). However, pupillometry showed an even smaller change of pupil diameter in imagery than in mental arithmetic, and the latter did not change acuity (Reeves & Segal, 1973). Moreover, when subjects imaged vertical lines (IM) or had no image (NI), the average Perky effect was large, 1.27 *d′* units, even when an artificial pupil was used to control gaze direction and obviate any changes in pupil size or accommodation of the lens due to imagery (Craver-Lemley & Reeves, 1987, Experiment 7). The optical effects suggested by Finke (1985) and others may occur, but are not primary causes of the Perky effect.

#### Distraction

Holding an image in mind might distract the subject from the visual task and lower sensitivity that way, as suggested by Dijkstra et al. (2021). However, in our acuity experiments, the Perky effect remained at full strength for 4 s to 5 s *after* the subject cleared away the image and could no longer be distracted by it (Craver-Lemley & Reeves, 1987, Experiment 7). Moreover, adding a physical distractor did not alter the Perky effect (Craver-Lemley & Reeves, 1992). Finally, the Perky effect goes to zero if the image is mentally projected behind the acuity target instead of on top of it (Craver-Lemley et al., 1997), even though any distracting effect should be the same. Distraction may occur, and if so may modulate, but clearly does not cause, the Perky effect.

#### Perceptual

Craver-Lemley and Reeves (1992) concluded that the Perky effect is not primarily due to optical distortion or distraction by the image, but rather, reflects a neural process. But what form might this take? Imagery could affect *d′* by influencing neural signals or noise, or both. Increasing noise or lowering signal would make it more likely that the signal and noise are confused by the detector, lowering *d′* and generating a Perky effect (Segal & Gordon, 1969). Reducing noise or increasing signal would create a ‘reverse Perky’ in which imagery increases *d′*.

The remainder of this paper is devoted to analyzing the Perky and reverse Perky effects to better pin down these possibilities. I will advance the thesis that imagery always enhances the signal, but also increases the noise, such that at low levels, signal enhancement dominates (producing the ‘reverse Perky’) and at high levels, interference dominates and the (regular) Perky occurs. In doing so I will rely on studies of visual interference since the auditory Perky effect has been so much less documented.

#### Response criteria

Too much (or too little) caution will shift the criterion away from the optimum (c = 0), making it more likely that the observer misclassifies signal as noise or noise as signal (Dijkstra et al., 2021; Farah, 1989). It was an important result of Segal and colleagues that the performance loss in imagery is primarily due to a depression of sensitivity, not to an inferior response criterion. However, Dijkstra et al. (2021) observed a small but significant change in bias, from c = + 0.51 in NI to + 0.29 when images were parallel to the target, but no change in c when they were orthogonal. In Segal and Fusella (1970, Table 1), the slope-adjusted biases c = + 0.85 in IM and c = + 0.55 in NI, changed in the opposite direction, and Reeves (1981) found no effect of imagery on bias using Yes/No. The model below addresses sensitivity but does not explain why the biases are little affected by imagery. One possibility, suggested by Gorea and Sagi (2000), is that subjects must learn the noise and signal distributions to optimize their criteria (i.e., get c close to 0). If such learning extends across conditions, it is impossible to maintain separate criteria in them. Running different subjects in NI and IM, or using block lengths long enough for independent criteria to develop, might determine whether imagery can, on average, substantially affect the criterion.

## Assumptions of TSD

The reader will have noticed how many assumptions have been needed to apply TSD to a behavioral experiment. To recapitulate, these are:

1. Independent sources of across-trial external noise and neural noise that sum
2. Noise is distributed according to a known function (e.g. Normal)
3. Signals have a fixed variance (or do not vary at all) across trials
4. Signals add to noise, shifting the probability distribution sideways
5. If the strength exceeds a criterion, the ‘decider’ reports Yes; otherwise No
6. Any within-trial variance can be safely ignored.
7. A sensory ‘detector’ is followed by an independent ‘decider’
8. Very few trials are pure guesses or lapses, and these can be discounted.

Some of these assumptions have been tested in the context of the Perky effect. Given d’s always below 3.0, as in Segal and Fusella (1970) and in our studies, Assumption 8 is reasonable. Assumptions 3, 5, 6, and 7 are intrinsic to TSD, not empirical (D. M. Green & Swets, 1966). Assumption 4 predicts a linear increase in *d′* with signal strength (D. M. Green et al., 1957; see below). Assumption 1 implies that *d′* varies inversely with the level of external noise, an experiment that still needs to be done. As discussed above, Segal and Fusella (1970) tested Assumption 2 by plotting the ROC curve in *z-*scores, as z(Phit) versus zP(fa), which is linear if the noise is normally distributed. This appears to be the only published test of this assumption. The reader should bear in mind that the application of TSD to the Perky effect is still somewhat tenuous, given this context.

## Signal level and *d’*: The current model

Elementary TSD predicts that sensitivity (*d′*) increases in proportion to the signal, S (D. M. Green et al., 1957), if the noise is constant. I will use the term **N**_**o**_ to refer to any constant intrinsic noise, independent of the presence of either the stimulus or any imagery. As before, N will continue to refer to the total noise, which may vary with the stimulus and with imagery. Equation 2 (below) assumes that N > **N**_**o**_, but Eq. 1 restricts noise to **N**_**o**_, so that N = **N**_**o**_. Thus:

1 $${d}{\prime}=\alpha S/{\mathbf{N}}_{\mathbf{o}}=\alpha S/\surd \left({{\sigma }_{No}}^{2}\right)=\alpha S$$

where α is a constant of proportionality. **N**_**o**_ is the square-root of the cross-trial noise variance, σ_No_^2^, so S and **N**_**o**_ are in the same units, and α and *d′* are unitless. If the noise level is unknown, then TSD takes the noise variance to be one, σ_No_^2^ = 1.0.

The noise in Eq. 1 is assumed to be physically equal in no imagery (NI) as in Imagery (IM). In this respect Eq. 1 is more specific that the usual TSD assignment of 1.0 to any unknown noise level, which has no further empirical meaning. Since increasing the signal by a has the same effect on *d′* as decreasing the noise by a, it may sometimes be convenient to fold a into the noise. However, Eq. 1 is written with **N**_**o**_ fixed. So α in NI, namely α_NI_, must be distinguished from α in IM—namely, α_IM_.

**Fig. 2:**
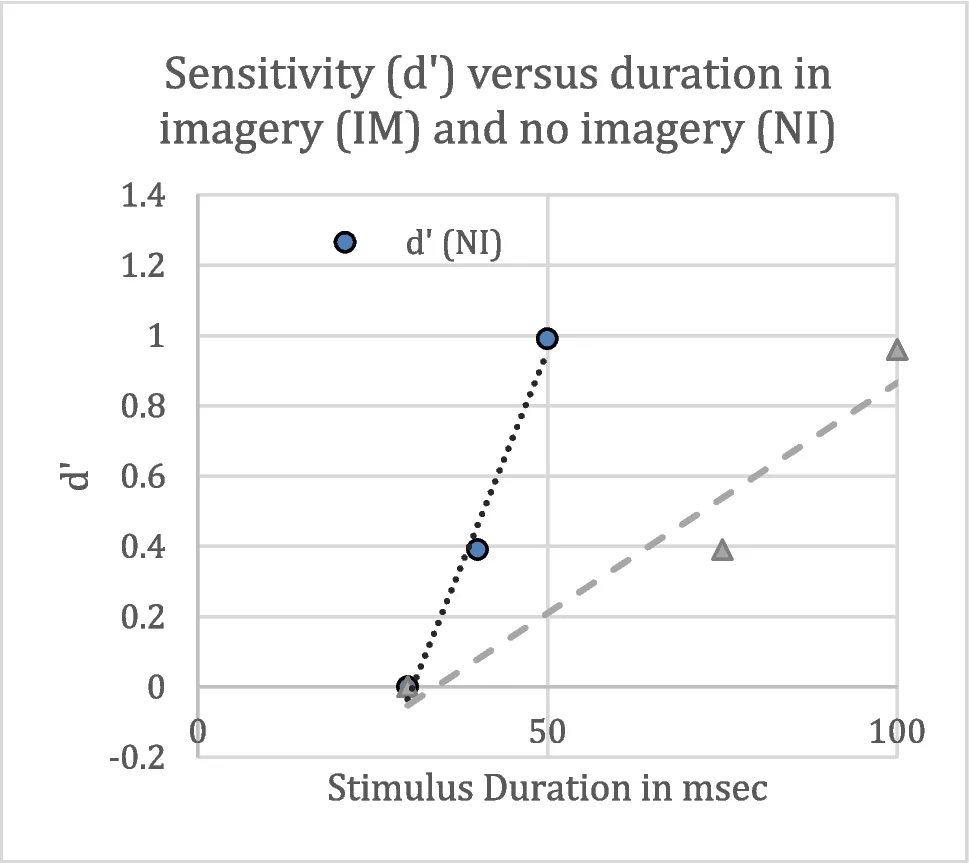
Sensitivity (*d′* in an 2AFC acuity task) versus stimulus duration in no-imagery (NI) and image vertical lines (IM), averaged across data from four well-practiced subjects in Craver-Lemley and Reeves (1987, Experiment 8a). The intercept at 30 ms is the duration below which the acuity judgement could no longer be performed

The Perky effect occurs if *d′*(NI)/*d’*(IM) > 1, which in terms of Eq. 1 implies that α_NI_/α_IM_ > 1 is independent of signal level, S. In a test of this ‘ratio rule’, Craver-Lemley and Reeves (1987, Experiment 8) obtained psychophysical curves for acuity targets presented in NI (no imagery) and IM (imagery; subjects imaged vertical lines surrounding the acuity target). Subjects reported if the lower line in the acuity target was left or right of the upper line; the position of the entire target was jittered across trials to force a relative judgement over one of absolute position. Mean *d′*s for four expert subjects are plotted in Fig. 2.

Recall that the signal, S, is a function of stimulus level, SL. To fit *d′*(NI), I assumed that SL = **t**, the stimulus duration in msec, and that S = **t** if 150 > **t** > 30 ms (McAnany, 2014) and S = 0 if **t** < 30 ms, the targets being indistinguishable below this duration. The data for the four experts plotted against** t** in Fig. 2 indicate *d′* linear with **t** in both NI and IM, with the slope in NI (α_NI_)—namely, 0.049, being 3.43 times the slope in IM (α_IM_)—namely, 0.0143. This ratio rule implies that *d′*(NI) and *d′*(IM) both increase with S, so the Perky effect, *d’*(NI) − *d’*(IM), must increase with *d’*(NI).

**Fig. 3:**
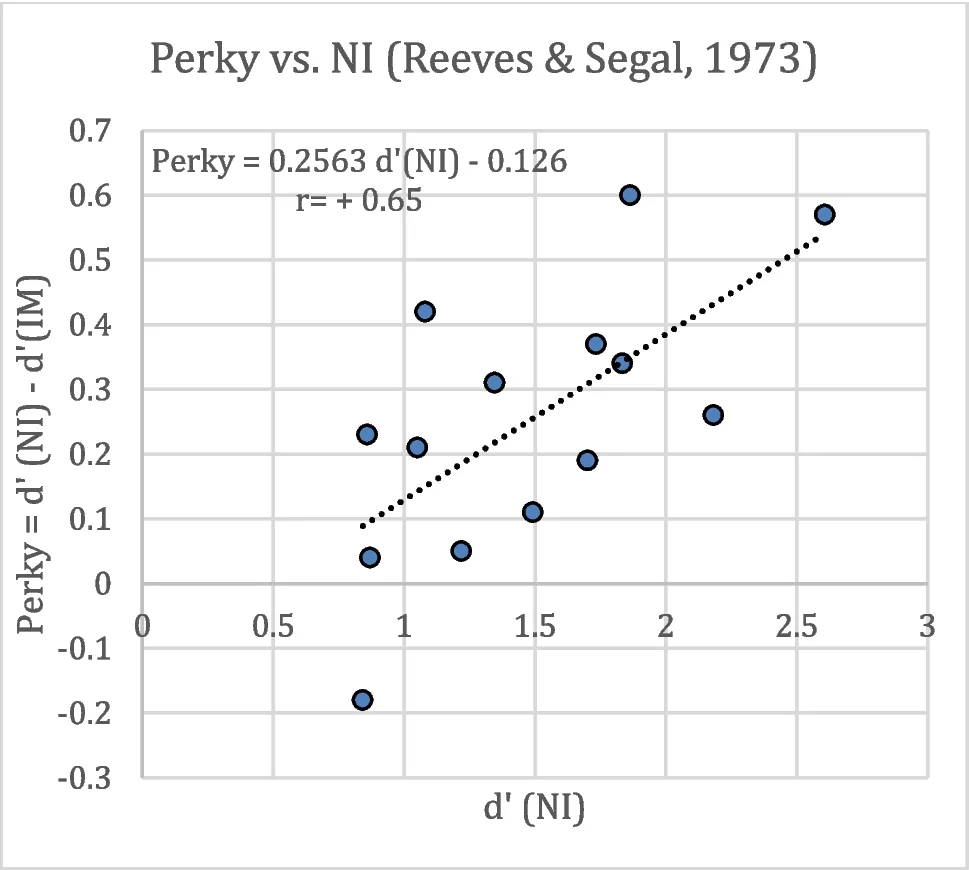
Individual Perky effects, *d′*(NI) – *d′*(IM), plotted against sensitivity in No Imagery, *d′*(NI). Data from 14 subjects in Reeves and Segal (1973). The Perky effect increased in subjects with higher performance. Criteria (not shown) did not differ significantly between NI and IM. One subject showed a ‘reverse Perky’ of 0.2 *d′* units, which we had overlooked

I reanalyzed some historical data to determine if the increase of the Perky effect at higher S levels was present in every study, as predicted by the ratio rule. The mean Perky effects of each of 14 subjects in Reeves and Segal (1973) show that it was (see Fig. 3.) Subjects created different images on each trial in this experiment, unlike subsequent experiments from our lab, but the increase still occurred.

**Fig. 4:**
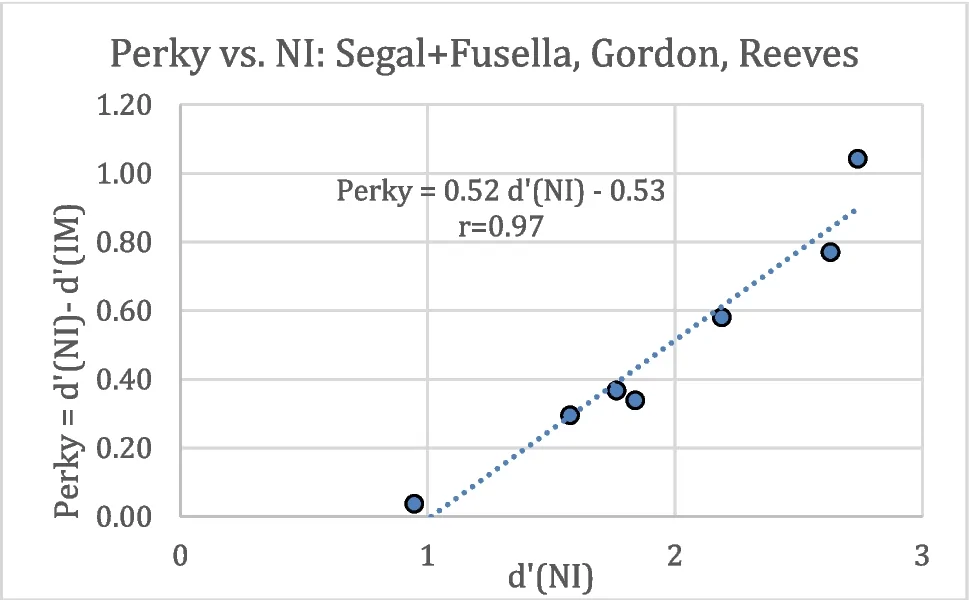
Mean Perky effects, *d′*(NI) – *d′*(IM), from four studies of the effects of visual imagery (IM) on vision, plotted against *d′*(NI). Means were taken from seven data sets, given in Segal and Fusella (1969) for 32 Ss in two groups; Segal and Fusella (1970) for 14 Ss; Reeves and Segal (1973) for 12 Ss in three groups; and Segal and Gordon (1969) for 12 Ss. The Perky effect clearly grows with *d’*(NI), the performance in no imagery. These means all come from separate groups of subjects, making it unlikely that this is an artefact of a single experiment or condition

Mean Perky effects in four studies by Sydney Segal are shown in Fig. 4. Each datum refers to the average over all trials and subjects in each study. These trials were all undertaken by projecting dim stimuli against the back wall of a hood made of translucent plastic. Since the Perky effects measured by tachistoscope, television, and with the hood, hardly differ (Reeves et al., 2020), I include these data here.

### The ‘reverse Perky’

Segal and colleagues had kept performance in NI at high levels so that the Perky effect would not be truncated at chance. However, for lower levels of performance, a ‘reverse Perky’ may occur, as first reported in terms of d′ by Ishai and Sagi (1995, 1997a, 1997b). These authors used very different stimuli from ours, namely Gabor patches, and we were concerned that the Perky effect might be stimulus dependent. In a larger study in our lab, again using acuity targets and images of vertical lines, we also found that *d′*(IM) exceeded *d′*(NI) at low levels (Reeves et al., 2020). We ran 124 subjects in a 2AFC acuity task, comparing IM with NI in four conditions of stimulation. We found a small ‘reverse Perky’ effect for scores averaging below 76% correct in 2AFC. Individual subjects crossed over from facilitation to interference at different levels of *d′*, but all those with a wide range of data did so. Since our results echoed those of Ishai and Sagi (1995, 1997a, 1997b) despite the change in stimuli, we concluded that the level of performance accounted for the reverse Perky.

**Fig. 5:**
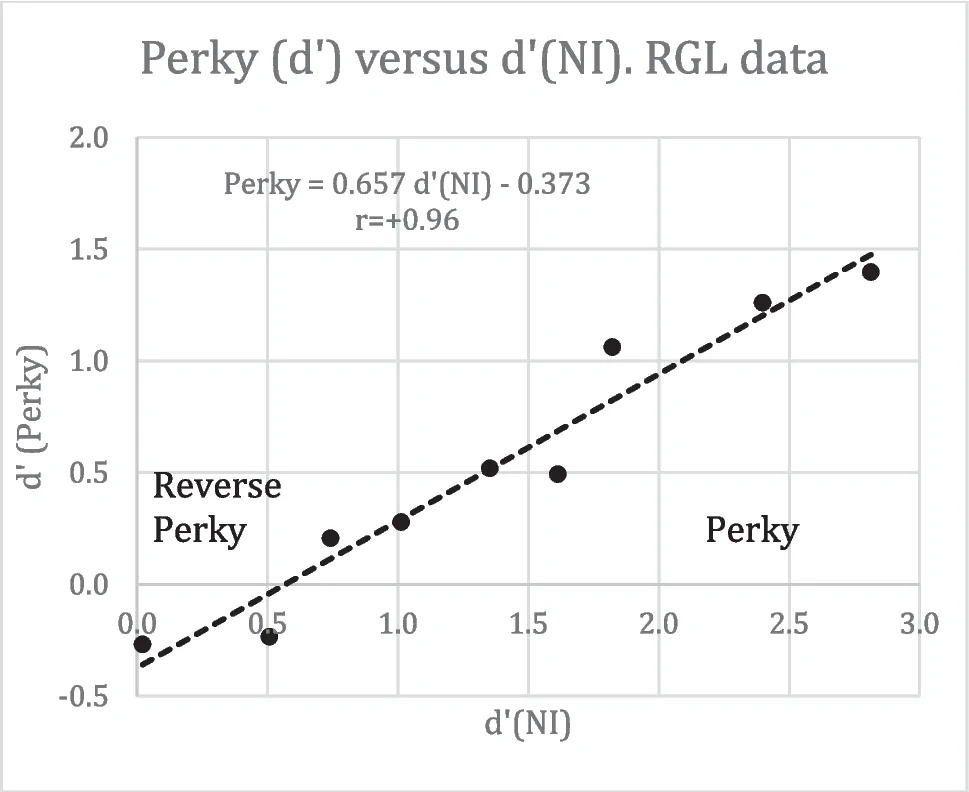
Re-plot of data from Condition A (2,000 ms, bright) in Reeves et al. (2020), showing the Perky effect in *d′* units as a function of mean *d′*(NI). Each dot shows an average over 16 participants with similar *d′*(NI) scores. The 32 individuals who scored poorly in NI showed the reverse Perky; those who scored better, the regular Perky. The increase in the Perky with *d′*(NI) illustrates the ratio rule

To illustrate, Fig. 5 replots the data from Reeves et al. (2020), Condition A (a 200-ms, bright target), converted from 2AFC accuracy to *d′*. Raw scores were grouped, 16 at a time, into nine bins. The lowest group (far left) averaged near chance in No Image, as *d′*(NI) = 0.02, but showed a striking ‘reverse’ Perky of − 0.27 *d′* units, as *d′*(IM) = 0.29, the negative values implying an *increase* in sensitivity in imagery. The top group (far right) had a typical regular Perky effect of 1.4 *d′* units. The linear regression fit well (*r* =.96). The estimated ‘break-point’ or crossover from reverse to regular Perky at *d′*(Perky) = 0 occurred at *d′*(NI) = 0.57. Of the four conditions run in that study, three revealed a clear-cut reverse Perky at low *d′*(NI) levels; in the fourth condition, the break point was higher and only two subjects showed a reversal. Note that Byczynski and D’Angiulli (2025), who ran 54 subjects at nearly subliminal levels on a 2AFC acuity task, also found a significant reverse Perky of *d′* = − 0.064 at a very low *d′*(NI) = 0.046. Thus the reversal at low levels has been verified on subjects run on acuity tasks in other labs, not just our lab, as well as those run with Gabor targets by Ishai and Sagi (1995, 1997a, 1997b), and with gratings seen in noise (Dijkstra et al., 2022; see below), so it seems secure.

## Experiment 1 (DMR)

### Method

In the current research, we hoped to obtain more extensive reversal data and also asked whether the reversal was affected by the type of image. We knew that the break point varied with stimulus duration and brightness (Reeves et al., 2020), but not whether image type had any effect. We compared *vivid* with *nonvivid* images which were either *static* (unmoving) or *dynamic*, following a suggestion by Amedeo D’Angiulli (personal communication, 2008). Our theoretical interest was in discovering whether Perky reversal is unique to the vivid, static images we and others have used, or is a general property of imagery.

#### Participants

Participants were 54 Northeastern undergraduates, over the age of 18 years, with normal or corrected-to-normal vision who received one hour credit for their participation. They were told that they were free to leave at any time during the hour, but none did. They were naïve to the purpose of the experiment but were debriefed afterwards. They were run after signing an informed consent form approved by the Department of Psychology and the Northeastern IRB board.

#### Stimuli and apparatus

Stimuli were presented on a white, 32 cd/m^2^, 15-in. ViewSonic 640 × 480 monitor, and viewed from 36 cm. An outline black frame centered on the screen and subtending 11° × 11° was presented during imagery. Participants were asked to form images that filled this frame as these tend to be created more quickly than those which fill smaller or larger frames (D’Angiulli & Reeves, 2007). On each trial a short vertical line was presented for 20 ms at left, middle, or right of screen center, the subject required to state which (i.e., a 3AFC task, chance being 33%.)

#### Imagery task

On a random half the trials, the ‘No Image’(NI) trials, subjects were requested to avoid imagery. (If imagery involuntarily occurred, the trial was re-run later on using the same stimulus.) On the remaining trials, images were either requested to be *static* (i.e., stationary) or to be *dynamic*, moving across the mind’s eye as far, and as fast, as the subject desired. Images were of 12 everyday objects (e.g., shoe, table, bird, hat) chosen to be easily imaged by most persons. Subjects were given 3 s to form each image. Each image was rated on a 4-point scale of vividness after each image trial, as follows: ‘very vivid’ (V1), ‘moderately vivid’ (V2), ‘vague and dim’ (V3), and ‘little or no image’ (V4), by pressing the corresponding labelled key.

#### Conditions

Static and dynamic images were blocked. Ratings were averaged over subjects and then split in two, with the top half of the ratings counted as ‘vivid’ and the rest as ‘nonvivid’. This defined four imagery conditions: static and vivid (SV), dynamic and vivid (DV), static and nonvivid (SNV), and dynamic and nonvivid (DNV). Vividness was not blocked as it varied from trial to trial.

### Results

**Fig. 6:**
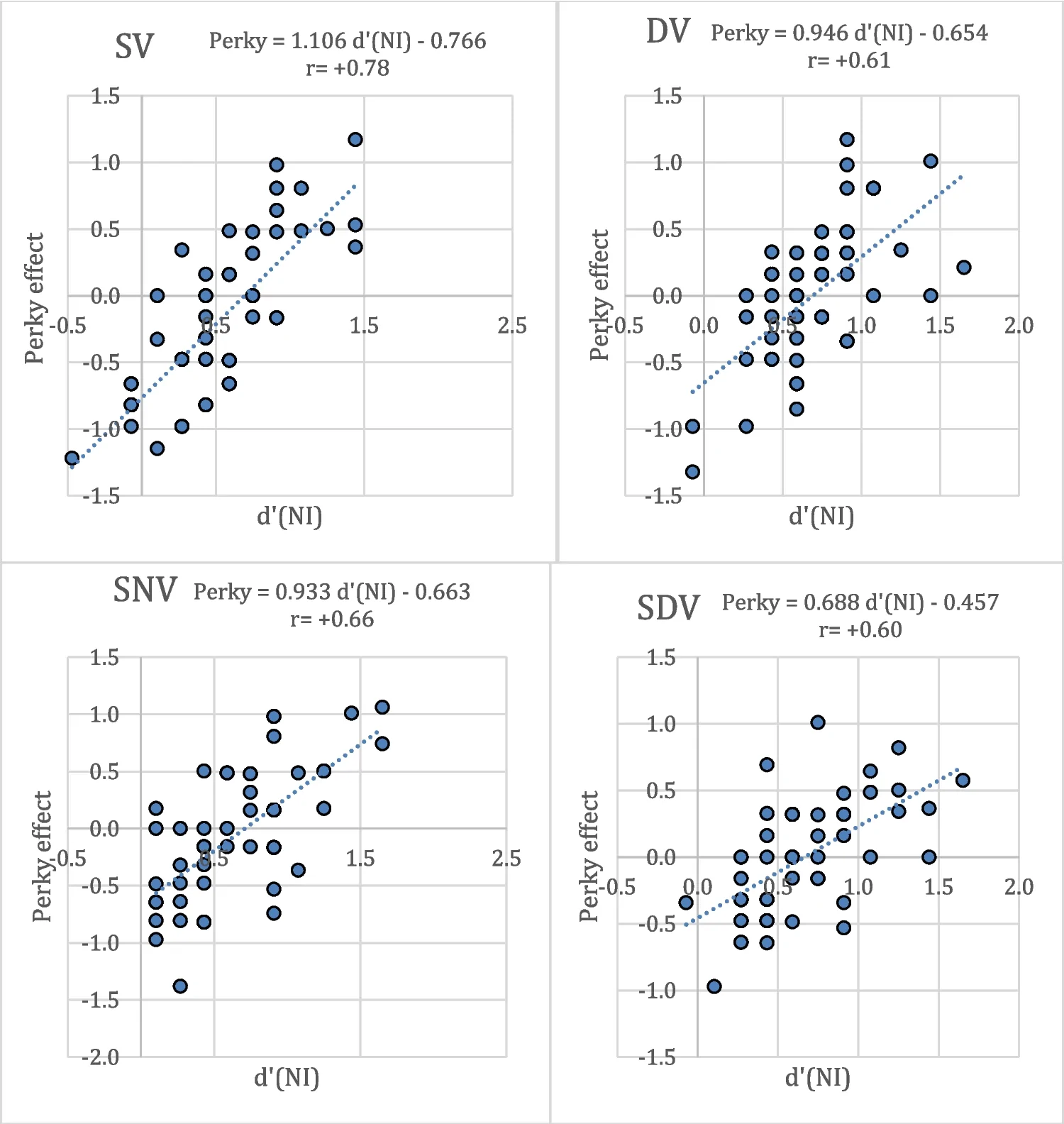
Data of Experiment 1 transformed from 3AFC accuracy to *d′* and plotted as Perky effects, *d′*(NI) − *d′*(IM), against *d′*(NI). Participants’ *d′*(NI) scores were grouped into 10 bins and fit by linear regressions. Scores to the left of the ‘break-point’, where *d′*(NI) = *d′*(IM), reveal the reverse Perky; those to the right, the regular Perky

Accuracy for reporting line location was converted to d′ using the formula of Smith (1982) for 3AFC. In the first analysis, participants’ d′ scores in NI were used to aggregate data into bins, ranging from *d′*(NI) < 0.1 up to *d′*(NI) > 0.8, in steps of 0.1. The number of observations in each bin ranged from 3 (to capture a few very high scores) to 20. Scatter plots of raw *d′*(Perky) against raw *d′*(NI) in each of the four conditions of the study, as shown in Fig. 6, show the changeover from the reverse or negative Perky for those who scored below about *d′*(NI) = 0.69 to the usual (positive) Perky for those who scored above this level.

Critically, the ‘break point’ or *d′*(NI) for which the Perky effect = 0 varied little with image type, being 0.69, 0.69, 0.71, and 0.66 for the static vivid (SV), dynamic vivid (DV), static nonvivid (SNV), and dynamic nonvivid (DNV) images. Moreover, the best-fit linear slopes relating the magnitude of *d′*(Perky) to *d′*(NI) varied only unsystematically from the mean slope of 0.92, being 1.11, 0.95, 0.93, and 0.69 respectively (SE_slope_ = 0.15).

**Fig. 7:**
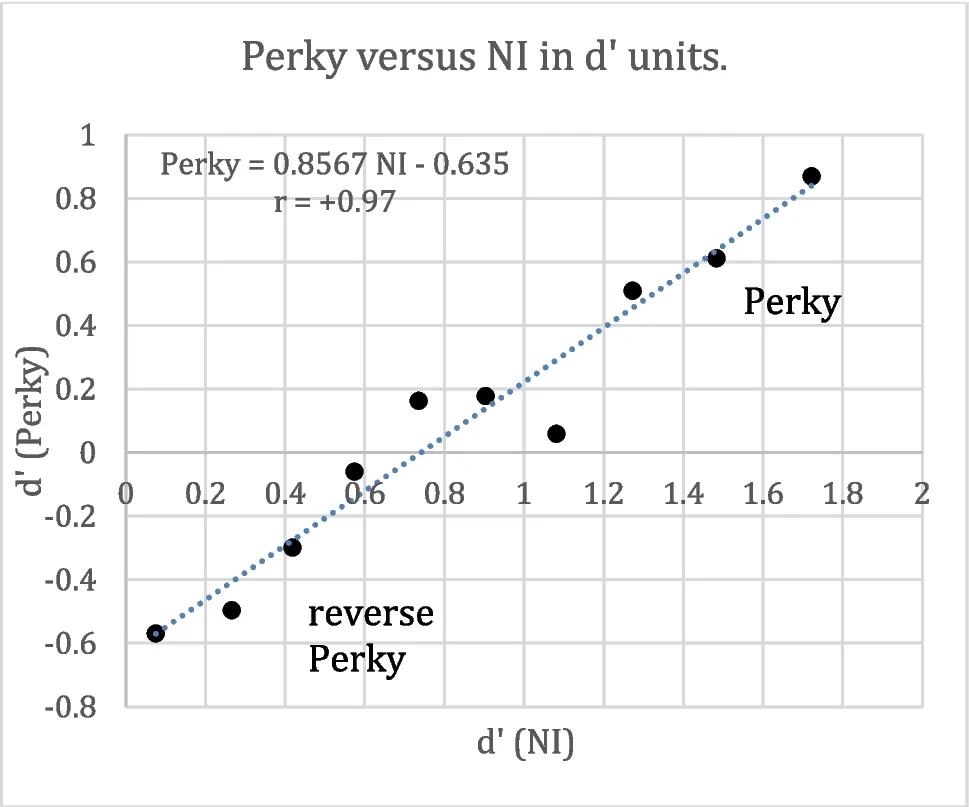
Data of Experiment 1 averaged over image type (see Fig. 6) and regrouped, 16 at a time, into 10 bins. The lowest group (far left) averaged near chance in NI, as *d′*(NI) = 0.08, but showed a striking ‘reverse’ Perky, since *d′*(IM) = 0.64, an *increase* in sensitivity due to imagery of 0.56 *d′* units. The top group (far right) had a typical Perky effect, a decrease in sensitivity of 0.9 *d′* units. The linear regression fit well (*r* =.97). The estimated ‘break-point’ or crossover from reverse to regular Perky was *d′*(NI) = 0.74

These data show little or no effect of image type on the Perky. We therefore collapsed across image type and rebinned the data into nine bins of equal increments in *d’*(NI), the number of scores in each bin varying from nine to 23. The slope of the linear regression between the Perky effect and *d′*(NI)was + 0.90; the correlation, *r* =.97 (*t*_8_ = 10.55, *p* <.01.) The reverse Perky occurred for participants scoring below the ‘cut-point’ of *d′*(NI) = 0.69, and the regular Perky occurred for those scoring above. To check whether binning by score biased this finding, each group of 16 successive *d′*(NI) scores were averaged into 10 bins of somewhat different widths. The linear regression was again significant (*r* =.97, *t*_9_ = 5.84, *p* <.01), the mean slope was 0.86, and the mean break-point was *d′*(NI) = 0.74, as shown in Fig. 7. The method of binning hardly changed the results.

### Discussion

To explain the original Perky data, we had assumed that the signal level, S, and the noise level, N, were uncoupled. The variance of the noise, σ_No_^2^, then remains constant when only the signal level is varied, as in Eq. 1. However, this cannot explain the cross-over from reverse to regular Perky. To do so, we assume that imagery boosts both the signal (by α_IM_) and the noise (by a factor, β). An expression for this relationship is provided by Eq. 2:

2 $${d}{\prime}={\alpha }_{IM} S/\surd \left[{\left(\beta {\sigma }_{S}\right)}^{2}+{{\sigma }_{No}}^{2}\right]$$

Here, the signal is defined to have mean S and variance σ_S_^2^ across trials. The total noise, N = β^2^σ_S_^2^ + σ_No_^2^, is then the sum of the usual additive noise **N**_**o**_ with fixed variance σ_No_^2^ = 1 and a statistically independent multiplicative noise with variance β^2^σ_S_^2^. The square-root converts the total noise into a standard deviation, so that *d′* is unitless. (If β = 0, Eq. 2 reduces to Eq. 1.)

Multiplicative noise is hardly novel. It was first considered by Swets et al. (1961) and has been discussed by Kontsevich et al. (2002), Solomon (2009), and many others. Equation 2 is similar to D. M. Green and Swets’s (1966) formula *d′* = S/√(σn^2^ + 0.5σsg^2^) for the case when the signal has variance σsg^2^. The innovation here is that the multiplicative noise depends on the presence of imagery. This is an empirical finding, not deduced from theory.

#### *Predicting mean *d′*s.*

The exact form of noise contributed by the signal has to be determined for the purposes of prediction. To be useful, Eq. 2 should preserve monotonicity; that is, *d′* must increases with S. To ensure that this is the case, the class of multiplicative noise must be specified. If this noise is exponentially distributed, as in ‘speckle noise’ in image processing (Goodman, 1976), then σ_S_ = S. In this case the monotonicity constraint is met by Eq. 2, since *d′* increases with S up to α_IM_/β. Multiplicative Poisson noise (σ_S_^2^ = S), or any other noise source with σ_S_^p^ = S, *p* > 1, is ruled out by monotonicity. With exponential noise, Eq. 2 predicts mean *d′* values in IM, just as Eq. 1 predicts mean *d′* values in NI.

#### Predicting variances

Given additive Normal noise and multiplicative exponential noise, the total noise distribution becomes one of a family of ex-Gaussians at each level of S, with the exponential tail lengthening in imagery at higher levels of S. This implies that the standard deviation of *d′*(IM), namely σ(IM), should exceed that of *d′*(NI)—namely, σ(NI), to an increasing extent at higher levels of S. More exactly, σ(IM)/σ(NI) must be proportional to the square-root of the ratio of variances, (S^2^β^2^ + **N**_**o**_) in IM and **N**_**o**_ in NI, which equals √(S^2^β^2^ + **N**_**o**_). Thus, Eq. 1 and 2 together predict that, for some constant k,

3 $$\sigma \left(IM\right)/\sigma \left(NI\right)=k\surd \left({S}^{2}{\beta }^{2}+{\mathbf{N}}_{\mathbf{o}}\right)$$

#### Defining strength

Recall that each researcher in the following studies used the same stimulus for every subject. The usual psychophysical TSD procedure is the contrary; *d′* is measured for each subject separately, over several levels of stimuli (e.g., D. M. Green et al., 1957). Linearity of *d′* with stimulus level SL or a transform of it, such that S = f(SL) for some monotonic function f, is the typical outcome. However, when *d′* varies among subjects all given the same stimulus, as in the next three studies modelled here, it is not obvious how to proceed. The way I chose was to employ the concept of an *effective stimulus* to define strength. The same stimulus is less effective for the low scorer and more effective for the high scorer. Assuming that Eq. 1 holds in the absence of imagery, S is the ‘effective strength’ such that α_NI_S = *d′*(NI). The reader should remember that the S axis here is no longer tied to an objective stimulus level, as when S = f(SL), but depends only on linearity (Eq. 1) and the measurement of *d′*(NI). The same consideration applies to Eq. 3. Ideally, σ(IM) and σ(NI) should be measured at each level of stimulation, and fit separately to each subject. Given the data at hand, I used the means across subjects of the within-subject standard deviations (see the next section).

#### Fits to the various experiments

**Fig. 8:**
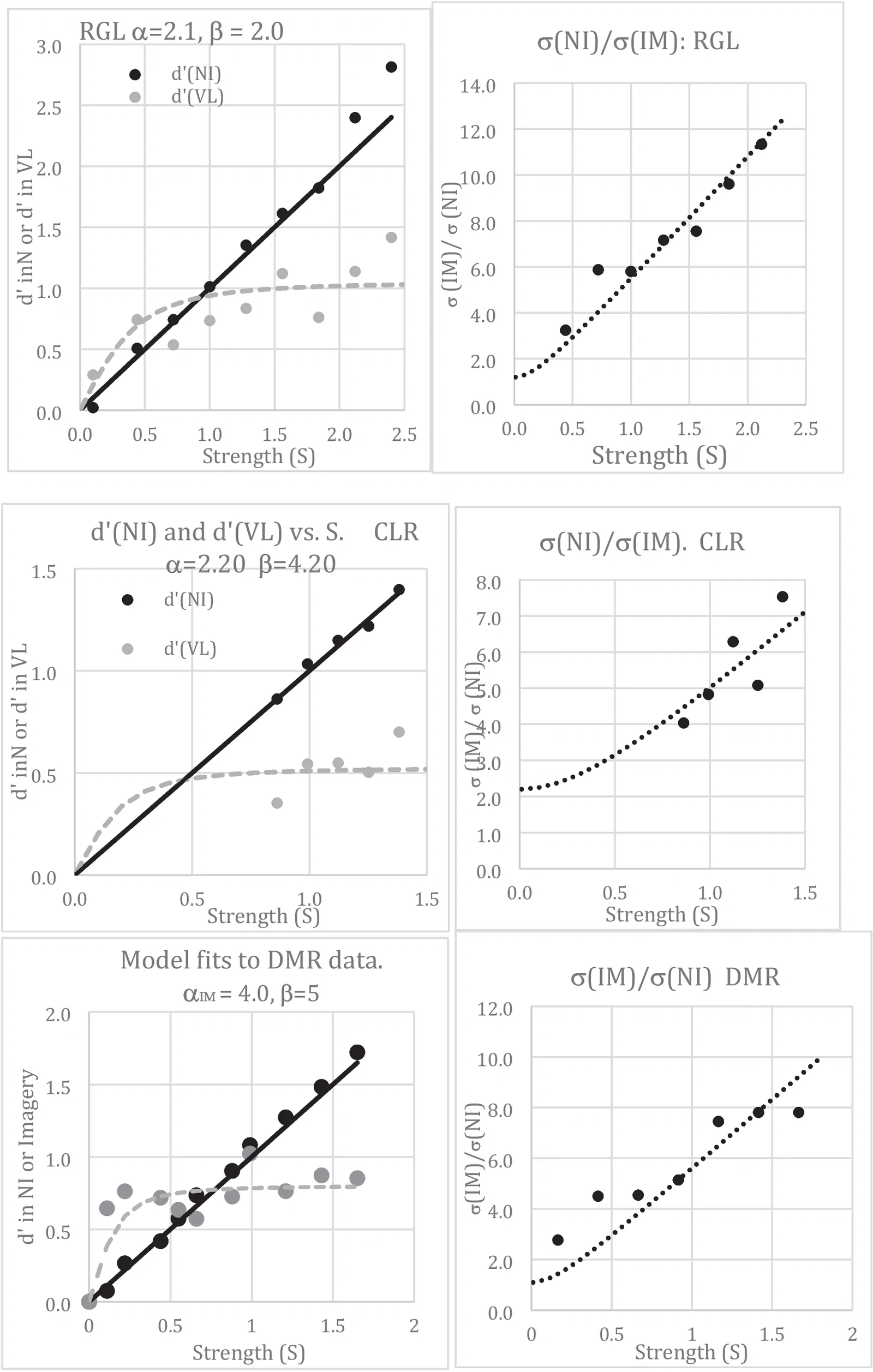
Left panels: *d′*(NI) (black circles), Eq. 1 (solid line), *d′*(IM) (grey circles) Eq. 2, (dotted line). Right panels: standard deviations of *d′*s in imagery, s(IM) divided by those in no imagery, s(NI) with the best-fit Eq. 3 (dotted line). Top: RGL data set (Reeves et al., 2020). Middle: CLR data set (Craver-Lemley & Reeves, 1985). Bottom: Current (Experiment 1) data set (DMR). See text for parameters

Figure 8 shows the least-square fits to each of three experiments, including the current one, with σ_**No**_ = 1. Exponential multiplicative noise σ_S_ = S was added to **N**_**o**_ in the denominator of Eq. 2. The fits to the mean d’s are on the left and to the s(IM)/s(NI) ratios on the right. In each case, raw *d′*(NI) values were ordered from least to most, and sorted into bins of 16 successive *d′*(NI) values with their 16 paired *d′*(IM) values. Mean *d′*s are in the left panels; black circles for NI, grey ones for IM, with model fits (black lines for NI, grey dots for IM) to the binned data.

Equation 2 was best fit (RMS) to *d′*(IM) with σ_IM_ and β free parameters. The ratios σ(IM)/σ(NI) of the standard deviations of each set of 16 ordered *d′*s in IM and NI are plotted against strength, S, in the right panels, along with the predictions of Eq. 3 with the same β as in the fit to *d′*(IM) and the best-fit k value (dotted lines). The range of strengths, S, was set arbitrarily from 0 to a maximum that was chosen for each experiment to match the range of *d′*(NI), so that a_NI_ = 1.0 in Eq. 1.

The fits to the RGL data (shown above in Fig. 5) are plotted in Fig. 8 (top). Setting S to range from 0 to 2.5, *d′*(NI) = S/**N**_**o**_ in Eq. 1, with α_NI_ = **N**_**o**_ = 1.0. Equation 2 was best fit to the paired *d′*(IM) scores with α_IM_ = 2.1, b = 2.0 (dotted line), although the fit is loose (root-mean square error, RMSE, being 0.17). The RGL data ratios σ(IM)/σ(NI) are plotted in Fig. 8 (top right) by black circles along with Eq. 3 (β = 2.0, k best fit to 2.5) (dotted curve) (*r* = +.97).

Raw data (CLR) were available to the author for 80 subjects from the thesis of Craver-Lemley (1985; reported by Craver-Lemley & Reeves, 1987). The middle-left plot in Fig. 8 shows the data, sorted and binned. S ranged from 0 to 1.5. Equation 1 best fit with α_NI_ = 1.0. Equation 2 was fit with α_IM_ = 2.2; β = 4.2. (RMSE = 0.09). Middle right: ratios σ(IM)/σ(NI) from the data. The dotted line plots Eq. 3 with k best fit to 1.10.

The current (Experiment 1) data set (DMR) is plotted in the bottom panels. S ranged from 0 to 1.65. Equation 1 was best fit with α_NI_ = 1.0. Equation 2 best fit with α_IM_ = 4.0, b = 5.0; the reverse Perky at low *d′*(NI) being clear-cut (RMSE = 0.11). Bottom right: ratios σ(IM)/σ(NI) from the data (black circles). The dotted line plots Eq. 3 with k = 1.15.

**Fig. 9:**
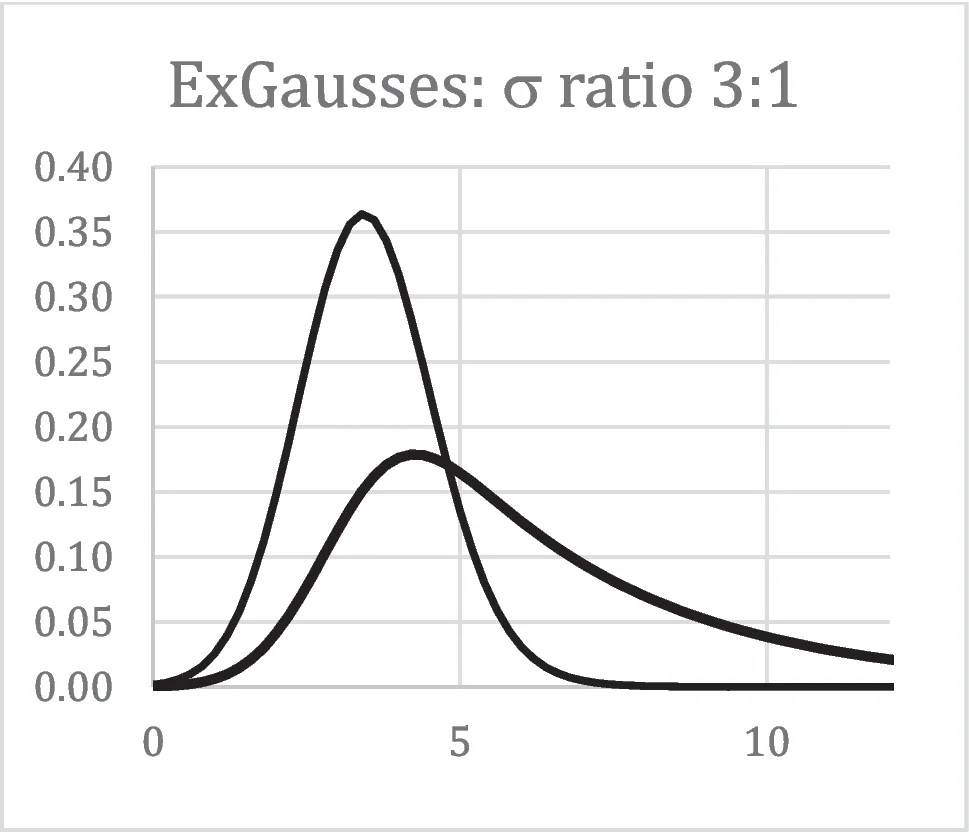
Illustrative ex-Gauss noise distributions as assumed in Eq. 2 (see text) for a low strength (little skew) and a high strength (skewed right.) The ss (1.17 and 3.5) are in the ratio 3:1. The *x*-axis is arbitrary. Since the noise distribution is assumed to be Gaussian in NI, with fixed s(NI), these curves imply a progressive increase in s(IM)/s(NI) with increases in S, as in the right-hand panels of Fig. 8

The multiplicative noise distribution in Eq. 2 depends on S. Figure 9 plots an ex-Gaussian noise distribution for low strength (S = 0.1) on the same axes as the ex-Gaussian for the highest strength in the DMR data (S = 1.65), for comparison. The s’s (1.17 and 3.5) are in the ratio 3:1. These represent the extremes of a family of ex-Gaussians, which become increasingly more skewed as S increases.

## General discussion

To recapitulate, the reverse Perky occurs for weak stimuli because the image boosts the signal by a_IM_ at all levels, but as S increases, bS increasingly contributes to N, limiting the growth in *d′*(VL) and generating the Perky effect. Equation 2 provides a simple description of this phenomenon, but to justify it functionally requires a logical reason why the presence of imagery adds stimulus-driven noise as well as enhancing the signal. This is discussed next.

### The observation window

Letting the signal increase the noise runs counter to the usual assumption in the theory of signal detection (TSD) that the noise source is uncoupled from the signal. Such uncoupling makes sense for an ideal spatio/temporal ‘observation window’, one which adheres tightly to the target in all conditions, since the noise inside the window is fixed. However, if imagery expands the observation window at higher levels, noise from the background will increase (e.g., Reeves et al., 1997), and this may explain the increase in stimulus-driven noise assumed in Eq. 2. Consequentially, if an external noise is imposed that is large enough to overwhelm bS, then the Perky effect should diminish, as found by Dijkstra et al. (2023) with high-contrast dynamic visual noise. A parametric study of the effect of added external noise is needed to test this idea.

An alternative to an expanded observation window is that subjects with higher levels of performance create more vivid images, which interfere to a greater extent. However, high vividness (VVIQ) is associated with a slightly smaller Perky effect, not a larger one (Craver-Lemley & Reeves, 1987). Interestingly, some individuals can match the vividness of their imagery to the contrast of an external stimulus (Reeves & Craver-Lemley, 2012). Matching might permit a test of this idea without having to rely on the VVIQ. Whether imagery expands the observation window or in some other way increases the noise is a question for future research.

### Psychophysical functions

The results of Dijkstra et al., (2022; Fig. 2) are of particular importance here, as the research was run impeccably, and as these authors measured psychophysical curves in both NI and IM. Their subjects either had no imagery (NI) or imagined gratings that were parallel (IMp) to or orthogonal (IMo) to a target grating. Targets were reported as present or absent (a Yes/No design). Target contrast, c, was 0 (no target) or was varied over six higher levels reported as a percentage of the noise mask level. The question at hand is whether Eq. 2 can describe their results. I read off P(‘Yes’)—that is, the chance of reporting ‘Yes’, from an expanded copy of their Fig. 2 at zero contrast (c = 0) and at three higher contrasts (c = 3.7%, 5.3%, and 7.3%). These rates are listed on the left hand-side of Table 1 for NI and the IMp and IMo images.

**Table 1:** Analysis of data from Dijkstra et al. (2022)

	Prob(‘Yes’)			*d′* = z(Phit)-z(Pfa)			Perky = *d′*NI − *d′*IM	
Rate	NI	Imp	Imo	NI	Imp	Imo	Imp	IMo
P(fa; c = 0%)	0.042	0.080	0.059					
P(hit; c = 3.7%)	0.218	0.345	0.210	0.95	1.01	0.76	− 0.06	+ 0.19
P(hit; c = 5.3%)	0.555	0.622	0.504	1.87	1.72	1.58	+ 0.15	+ 0.29
P(hit; c = 7.3%)	0.857	0.891	0.782	2.80	2.64	2.34	+ 0.16	+ 0.45

**Fig. 10:**
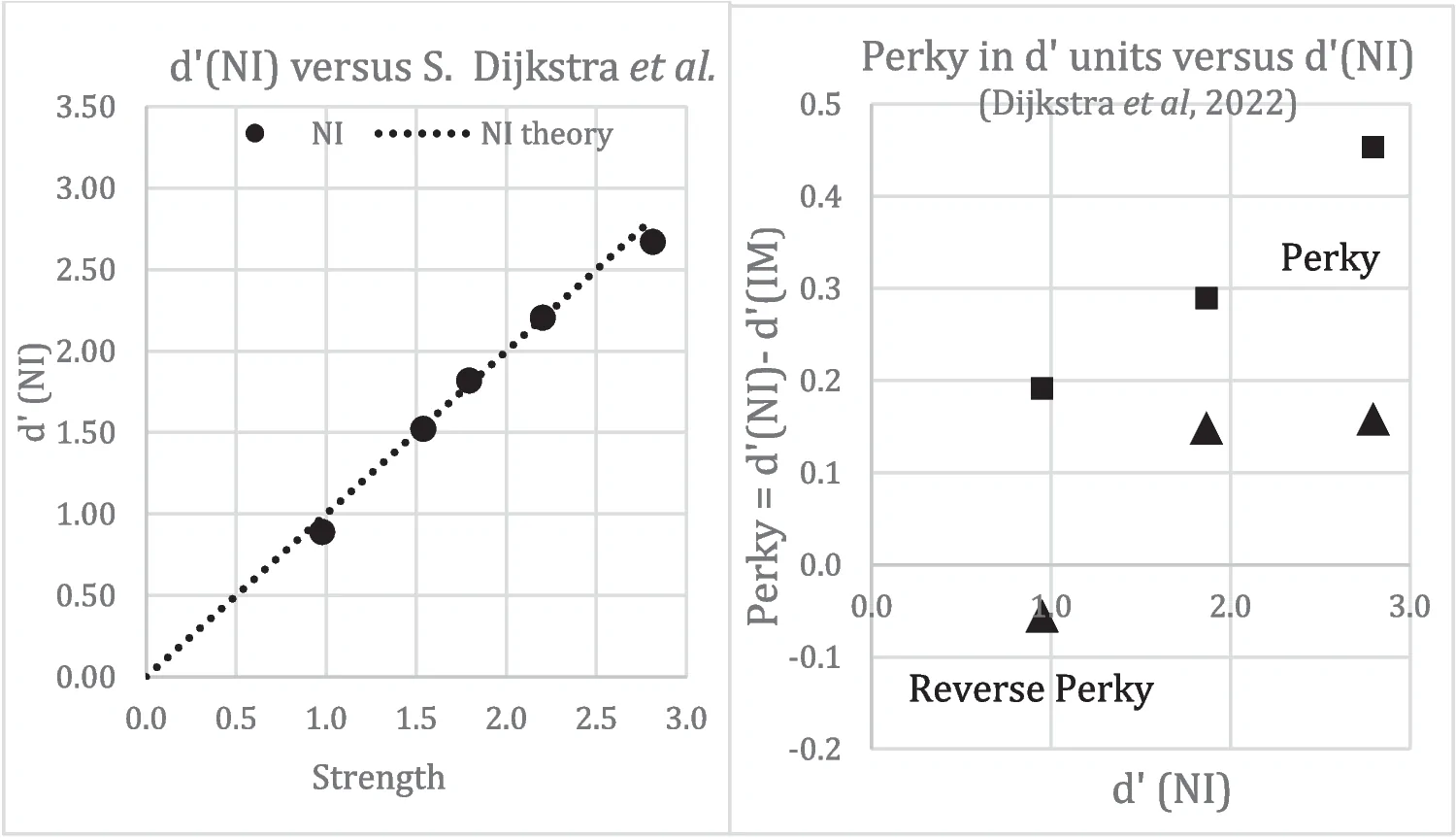
Replot of data from the psychophysical study of Dijkstra et al., (2022; Fig. 2) in terms of *d′*. Left panel: increase of *d′*(NI) with strength. Right panel: Perky effects, *d′*(NI) − *d′*(IM). Note the compressed y-axis scale, as both the Perky effect and the reverse Perky were reduced, possibly because of the added noise from the external mask (see text)

Since responding Yes at c = 0 defines no target, i.e. a false alarm, while responding Yes at higher contrasts defines hits, it is possible to convert the response rates to *d′*s (middle of Table 1) to find Perky effects, *d′*(NI) − *d′*(IM) (right side). This revealed a reverse Perky of − 0.06 *d′* units at the lowest contrast in IMp, but regular Perky effects of 0.15 and 0.16 at the higher contrasts (Fig. 10, right panel, black triangles). For image IMo, only Perky effects occurred, of 0.19, 0.29, and 0.45 at the three increasing contrasts (Fig. 10, black squares.) These Perky effects show the same pattern as in Fig. 8, above, with the ‘break-point’ for IMp at *d′*(NI) ~ 1.2.

Dijkstra et al. (2022) had reported seven contrast levels, c, including c = 14% where there were essentially no errors. After converting to *d′* units, the *d′*(NI)s, excluding the 14% datum, were linear with contrast, as required by Eq. 1; *d′*(NI) = 0.51c − 0.91 (*r* = + 0.99). These *d′*(NI)s are plotted in Fig. 10, left panel, against strength, S, with S = f(SL) = (0.51c − 0.91). This transform ensures that *d′*(NI) is linear with S and passes through the origin (*d′* = 0 when S = 0) as required by TSD. The additive term is required because target contrast is not absolute but defined relative to the mask.

Dijkstra et al. (2022) only considered the hit rates, not *d′*s, as they were interested in a theory about the subject’s criteria. However, as five of six of their *d′*s showed Perky effects, I dispute their conclusion that ’These results suggest that imagery adds sensory evidence to perceptual signals, thereby increasing the visibility of perceived stimuli.' (Dijkstra et al., 2022), as suppression occurred at almost all levels in this experiment. True, their Perky effects on average are considerably smaller than in the work of Segal, Craver-Lemley, Reeves and colleagues, even at high *d′*(NI) levels (Fig. 10, right panel). This can be explained in terms of Eq. 2 if the external noise from the masking pattern overwhelmed the multiplicative noise term, βS. To test this, best-fits to *d′*(IM) with Eq. 2 were obtained with α_NI_ = 1, σ_IMp_ = 1.05, σ_IMo_ = 0.88, and β = 0.05 in IMp and 0.02 in IMo (RMSE = 0.11). Thus βS < < **N**_**o**_ = 1 at every level of S, as hypothesized.

Since b was so small, the noise distribution should approximate a Gaussian even at high levels of S. Thus, the ratio σ(IM)/σ(NI) should not noticeably increase at higher contrast, as one may verify in Fig. 2 of Dijkstra et al. (2022). This supports Eq. 3 but only in a negative way; for proof, one would need to remove the masking noise and permit bS to be substantial for the same subjects and target stimuli. A systematic study of the effects of varying external noise in both NI and IM still needs to be done.

### Effect of orientation

Craver-Lemley and Reeves (1987) had showed that the Perky effect (at high levels) was insensitive to the relative orientation of the image and the acuity stimulus, whereas Sagi and Ishai (1995), who first reported the reverse Perky, found a strong effect of orientation; only parallel images, not orthogonal ones, aided detection of a near-thresholds vertical Gabor grating. This is also seen in the above analysis of Dijkstra et al.’s (2022) data, where the reverse Perky only occurred for the parallel image, IMp. These results can be explained in the context of Eq. 2 if images boost similar (‘congruent’) stimuli (α_IM_ > 1) but not dissimilar ones (α_IM_ = 1).

To explain why this might be, Reeves et al. (2020) borrowed Foley’s (1994) idea that an accelerating non-linearity which amplifies responses to similar stimuli (such as masks and tests of similar orientations) at low contrasts is followed by a divisive normalization at high contrasts which pools over surrounding cortical cell responses, regardless of orientation, to protect from saturation. Some version of this idea may ultimately justify Eq. 2, although the numerator would need modification to incorporate any accelerating nonlinearity. However, as it stands, Eq. 2 is purely formal and is not based on current neural modelling or physiology.

### Reduced Perky over time; an artefact

As many authors have pointed out (e.g., Byczynski & D’Angiulli, 2023; Dijkstra & Fleming, 2023), the reported Perky effects have diminished over the decades since Segal’s pioneering studies in the 1960s. Grayhem (2007) tested a possible explanation for this, based on C. S. Green and Bavelier’s (2007) discovery that playing recently developed video games improves acuity. Her subjects reported how often they played video games, how many hours they played in one sitting, how many per week, and how many years they have been playing. None of these factors influenced the Perky effect, perhaps because video games improve acuity equally in NI and imagery. We had also wondered if the use of monitors rather than tachistoscopes reduced the Perky effect, but Reeves et al. (2020) found no difference between the Perky effects of students tested on a television monitor and those tested in a tachistoscope on equated stimuli. Those studied have not changed; all the data have been collected on young university students since 1964. The better explanation is that psychophysical methods have been increasingly used in which mean accuracy in NI is around 71% (as in Ishai & Sagi, 1995, 1997a, 1997b), or lower, rather than the 90% used by Segal and by us. Lowering accuracy in NI brings participants’ scores closer to the break-point, so the overall Perky effect is reduced by averaging in those who score poorly, and this has happened to an increasing extent as the decades have passed by. In fact the top-scoring subjects in NI, as shown in Figs. 2, 4, 5, and 7, reveals the same healthy Perky effect (above 0.8 *d′* units) with little change across the several decades. We conclude that the apparent reduction in the Perky effect is an artefact of changes in psychophysical method.

### Theories of imagery–perception interaction

I now depart from the minutia of the Perky effect to consider the larger picture. There are two major contrasting theories of neural-visual interactions. One may be termed ‘semantic-associative’, the other, ‘modality-driven’. According to the *semantic* view, representations (i.e., cognitions conveyed by words or signs, associated memories or thoughts) can penetrate the visual system and in some sense ‘tune’ it. The concept ‘apple tree’ may help aid search for an apple, may help the sensory pathway that detects relevant apple features, and may evoke a concrete multi-sensory representation (in this case, tactile, taste, color, and shape of an apple), independently of the lighting, the viewing angle, and distance to the apple. In contrast, the *modality-driven* theory emphasizes visual variables such as proximity, color, contrast, and image-percept similarity. Meaning is only important in guiding the observer as to what to imagine; but once an image is created, how it interacts with perception is determined at a sensory level.

Currently, there is strong evidence that visual factors do affect image-perception interactions, and weak evidence that semantic associates do not. Thus, the magnitude of the Perky effect, 0.8 *d′* units, was the same for a block of trials with highly varied visual images (trees, boats, shoes, etc.) on each trial as for a block with unvarying images of vertical straight lines (Reeves & Segal, 1973). In Experiment 1, above, type of image had no effect on the break-point and at best a marginal effect on the slope (Fig. 3). In contrast to this null semantic effect, increasing the spatial distance from the visual stimulus (an acuity target) to an image of surrounding lines from 0.2° to 1.6° of visual angle reduced the Perky effect from 0.8 *d′* units to 0.2 *d′* units (Craver-Lemley & Reeves, 1987). When observers imagined both close and distant lines together, the Perky effect was also much reduced (Reeves & Craver-Lemley, 2012), as if the distant imagined lines had unmasked the close ones, an effect found in studies of visual masking. The two theories are not rivalrous; in principle, sensory and semantic factors may play a role, but the ‘modality-driven’ theory is, in our hands, much solider.

A version of the modality-driven theory, which may be termed modality-specific, was first proposed by Segal and Fusella (1970). They tested the detection of visual and auditory stimuli in conditions of No imagery (NI), Visual Imagery, and Auditory Imagery. The stimuli were fixed (a small blue arrow and a harmonic chord in their Experiment 1; three parallel green bars and a pure tone of 250 Hz in Experiment 2), but the images varied over trials (e.g., a bell ring, a dog barking, the sound of a mechanical typewriter; seeing a volcano, a table). Averaging *d′*s over 14 subjects from Experiments 1 and 2, sensitivity was high in NI in both modalities, averaging *d′* = 2.7 (Table 2, first row). Imagery in the same modality as the stimulus dropped *d′* to about 1.7, for a within-modality Perky effect of *d′* = 1.04 (Vis) and 0.94 (Aud), as shown in Table 2 (last row.) However, the *cross-modality* Perky effects were also considerable, namely *d′* = 0.61 when auditory images interfered with vision, and *d′* = 0.49 when visual images interfered with hearing (penultimate row). This was downplayed by Segal and Fusella, but it does cast doubt on the modality-specific hypothesis.

**Table 2:** Mean *d′*s (14 Ss): Segal and Fusella (1970)

	*d′*:Stim-Vis	*d′*:Stim-Aud
No Image (NI)	2.74	2.72
Image Vis	1.70	2.23
Image Aud	2.13	1.78
Perky-cross	0.61	0.49
Perky-within	1.04	0.94

One way to rescue modality-specificity is to assume that ‘cross modality’ interference is merely a symptom of distraction by the image, since each new trial in Segal and Fusella (1970) called for a new image, presumably requiring extra attention. By requiring the same image on every trial, we (Craver-Lemley & Reeves, 1987) showed that the postulated distraction effect could be reduced to about 0.20 d′ units. Moreover, Craver-Lemley and Reeves (1992, Experiment 7) found no effect of auditory imagery on visual acuity when we used the same set of mental auditory images (e.g., horns, bells) as had Segal and Fusella (1970). However, if distraction did account for the cross-modal Perky effects in Segal and Fusella (1970), then the pure modality-specific Perky effects would be *d′* = 1.04 − 0.61 = 0.43 in vision and *d′* = 0.94 − 0.49 = 0.45 in audition (Table 2), much less than expected given their high *d′*(NI) level (2.7). So, their cross-modal effects may transcend distraction. Further research is required to establish the limits and stimulus dependence of any cross-modal imagery interference. Visual acuity, our favored task, may be a special case which almost entirely excludes cross-modal effects.

To the extent that modality-specificity holds up, as in acuity, one can infer from the general properties of the visual system that the temporal and spatial extents of imagery (its ‘spread’) must be larger than the minimum visible at each eccentricity, since the minimum visible depends on tiny receptive fields (RFs), and imagery-spread depends on feedback from images generated in higher-level visual object areas with larger RFs. How such feedback due to imagery is instantiated makes difference. Craver-Lemley and Reeves (1992) speculated that in imagery, LGN neurons at the stimulus location were attenuated by feedback from higher cortical levels to protect the mental image from sensory interference. They drew this conclusion because image-target orientation (at high levels) had no effect on *d′*, and the LGN is the last stage in the visual pathway to be insensitive to orientation. However, some other mechanism must account for the orientation-sensitive reverse Perky found at lower levels found by Sagi and Ishai (1995) and Dijkstra et al. (2022). Moreover, all this may only apply to larger scale stimuli (say, gratings, or well separated acuity lines) where the somewhat coarser RFs involved in generating imagery can match the larger RFs needed to see the stimulus. No-one has tested the Perky effect with hyperacute stimuli.

Interestingly, one study (Reeves, 1981) required subjects to report if a much *larger* target, 5.8° across, was in the shape of a square or a triangle, in NI or IM. The target was presented dimly on a white field for 1.5 s (Segal & Fusella had used 2-s presentations to demonstrate the Perky effect). Red targets showed a nice Perky effect of 0.85 *d′* units, in both Y/N and 2AFC, but white targets did not (0.2 in FC and 0.0 in Y/N) even though *d′*(NI) was high (2.0). Sensitivity was the same whether subjects imaged a square, a bit larger than the target, or imaged various other objects, different on each trial, so it seems unlikely that the failure to find a Perky effect with white-on-white targets was due to a failure of imagery. Reeves (1981) concluded that white targets on white fields stimulated a luminance-sensitive channel which did not suffer from imagery interference, but this was falsified by Craver-Lemley and Reeves (1987), who found strong Perky effects with black targets on white fields. In Eq. 2, the Perky effect is minimal with b < < **N**_**o**_, so the white-on-white data do not necessarily rebut Eq. 2, but this is entirely post hoc.

Sagi and Ishai (1997b) had reported that, at the same level of performance, images recalled from long-term memory can hinder detection of Gabor targets, illustrating a Perky effect, whereas images created just 50 trials before target onset (and thus present in what they terms ‘short-term’ memory, STM) can facilitate detection (‘reverse Perky’). It is possible that their subjects were close to the ‘cut-point’ as their subjects’ 2AFC accuracy of 71% in NI implies *d′*(NI) = 1.1. Similar performance in NI and IM implies that S is the same, so their memory effect must be carried by α_IM_ or b in Eq. 2; for example, α_IM_ might be larger in the STM than in the LTM condition due to recency. A psychophysical study in which stimulus level was varied in the two conditions could ascertain whether this was the case.

### Origin of imagery–vision interactions

Assuming ‘modality-specificity’, one can hypothesize that imaginary visual patterns modulate lower-level perceptual entities in a top-down fashion, and that this modulation occurs in visual cortex (e.g., V1–V2; Cui et al., 2007) and associated areas (e.g., LOC), but not in the higher cortical regions involved in meaning or semantic relations. Evidence that imagery affects striate cortex (V1 and V2) is strong (Kosslyn et al., 1999). The visual properties of visual images are generated in higher-level visual object areas in the right hemisphere (Farah et al., 1985). Dijkstra and Fleming (2023) found that the pre-supplementary motor area, anterior insula, and right dorsolateral prefrontal cortex are also involved in image generation. However, for intermediate extra-striate visual areas, such as those involved in color constancy or crowding (V4), motion perception (MT, MST), the evidence is missing. There have been no studies of the direct effects of moving visual mental images on motion perception, as far as I know, and only the present study compares the effects of static and moving images, so there are too few facts about dynamic imagery to test against theory. The studies that do exist are explorations of the effects of dynamic imagery practice on subsequent performance (e.g. in basketball). It is not known whether the ongoing perception of moving bodies is enhanced, or suppressed, by concurrent imagery.

A further factor to consider is the possible energizing effect of having an image, which may activate pattern-insensitive sub-cortical structures such as the amygdala or the ascending reticular activating system important for dream imagery. In the comparison of NI with IM made in the current paper, the assumption has been that activity is equal in the two conditions, so only the pattern made by the image, not its mere presence, is relevant. That the Perky effect decreases to 0.2 *d′* units as the distance from the acuity target to the imagined vertical lines increases to 1.6° (Craver-Lemley & Reeves, 1987), that the effect is reduced from 15 to 10% if more lines are added to a close-in image to ‘unmask’ it (Reeves & Craver-Lemley, 2012), and that the effect disappears if the image is mentally projected behind rather than in front of the target (Craver-Lemley et al., 1997), all indicate that the pattern, not the overall activity level, matters. Will this still hold true if tested with complex, realistic visual imagery?

## Data Availability

Raw data and analysis are available from the Author on request.

## Code availability

Raw data, analysis, and equation fitting are all available in an Excel file, obtainable by request to the author.

## Declarations

### Open practices statement

The data of Experiment 1 and the analysis are available as an Excel file upon request to the author. Experiment 1 was not registered in advance.
