# Correction to: Imagery: interference, facilitation, and theory

**DOI:** 10.3758/s13414-026-03304-w

**Published:** 2026-07-28

**Authors:** Adam Reeves

**Affiliations:** Department of Psychology, Northeastern University, Boston, MA 02115 USA

**Correction to: Attention, Perception, & Psychophysics**

10.3758/s13414-026-03251-6

Due to misunderstandings by the typesetter, the Greek letters (α, β, σ) were replaced by Roman ones (a, b, s), and the subscripts were removed, in the body of the text. Equations 1, 2,and 3 were set correctly, so they no longer corresponded to the text. The corrected text is as follows:

1. Page 5, line 52 to Page 6, line 10: the various variances σ^2^ were typeset as s^2^
2. Page 7, line 13 -17. (In section: *d’ and c with noisy signals.* )
  If the signal is constant across trials, the signal plus noise distribution, S+N, has the same variance as N, so σs= σn, and the slope, s, of the ROC curve will be 1.0. If S fluctuates across trials, with variance σsg^2^, then the S+N distribution is wider with variance σs^2^=σsg^2^+σn^2^, and the ROC slope is s=σn^/^σs=1/σs, given unit variance noise, σn=1.
3. Page 10, lines 19-21. (Immediately following Eq. 1):

where α is a constant of proportionality. **N**_**o**_ is the square-root of the cross-trial noise variance, σ_No_^2^, so S and **N**_**o**_ are in the same units, and α and d’ are unitless. If the noise level is unknown, then TSD takes the noise variance to be one, σ_No_^2^ =1.0. . However Eq.1 is written with **N**_**o**_ fixed. So α in NI, namely α_NI_, must be distinguished from α in IM, namely α_IM_.

Page 10, lines 27-28.

However Eq.1 is written with **No** fixed. So α in NI, namely α_NI_, must be distinguished from α in IM, namely α_IM_. The Perky effect occurs if d’(NI)/d’(IM) >1, which in terms of Eq. 1 implies that α_NI_ / α_IM_ >1 is independent of signal level.

Page 10, lines 44-46

The data for the 4 experts plotted against** t** in Fig.2 indicate d’ linear with **t** in both NI and IM, with the slope in NI (α_NI_), namely 0.049, being 3.43 times the slope in IM (α_IM_), namely 0.0143.

(4) Page 17,lines 2-8. Section : DISCUSSION

To explain the original Perky data, we had assumed that the signal level, S, and the noise level, N, were uncoupled. The variance of the noise, σ_No_^2^, then remains constant when only the signal level is varied, as in Eq. 1. However, this cannot explain the cross-over from reverse to regular Perky. To do so, we assume that imagery boosts both the signal (by α_IM_) and the noise (by a factor, β).

(5) Page 17: lines 14-19.

Here, the signal is defined to have mean S and variance σ_S_^2^ across trials. The total noise, N=β^2^σ_S_^2^+σ_No_^2^, is then the sum of the usual additive noise **N**_**o**_ with fixed variance σ_No_^2^ =1 and a statistically independent multiplicative noise with variance β^2^σ_S_^2^. The square-root converts the total noise into a standard deviation, so that d’ is unitless. (If β=0, Eq. 2 reduces to Eq. 1.)

(6) Page 17: lines 23-24

Eq 2 is similar to Green & Swets (1966)’s formula d’=S/√(σn^2^+0.5σsg^2^) for the case when the signal has variance σsg^2^.

(7) Page 17: lines 33-37

.. (Goodman, 1976), then σ_S_=S. In this case the monotonicity constraint is met by Eq 2, since d’ increases with S up to α_IM_/β. Multiplicative Poisson noise (σ_S_^2^=S), or any other noise source with σ_S_^p^=S, p>2, is ruled out by monotonicity. With exponential noise, Eq. 2 predicts mean d’ values in IM, just as Eq. 1 predicts mean d’ values in NI.

(8) Page 17, lines 44-49 (in section *Predicting variances*)

This implies that the standard deviation of d’(IM), namely σ(IM), should exceed that of d’(NI), namely σ(NI), to an increasing extent at higher levels of S. More exactly, σ(IM)/σ(NI) must be proportional to the square-root of the ratio of variances, (S^2^β^2^+ **N**_**o**_) in IM and **N**_**o**_ in NI, which equals √(S^2^β^2^+ **N**_**o**_).

(9) Page 18, lines 10-11. Assuming that Eq. 1 holds in the absence of imagery, S is the ‘effective strength’ such that α_NI_S=d’(NI).
(10)  Page 18, line 15. Ideally, σ(IM) and σ (NI) should be measured…
(11)  Page 18, line 22. with σ_**No**_=1. Exponential multiplicative noise σ_S_=S was added to **N**_**o**_ in the denominator of Eq 2.
(12)  Page 18, lines 29-33.

Eq 2 was best-fit (RMS) to d’(IM) with α_IM_ and β free parameters. The ratios σ(IM)/σ(NI) of the standard deviations of each set of 16 ordered d’s in IM and NI are plotted against strength, S, in the right panels, along with the predictions of Eq 3 with the same β as in the fit to d’(IM)

(13)  Page 18, lines 38-44 The fits to the RGL data (shown above in Fig. 5) are plotted in Fig. 8 (top). Setting S to range from 0 to 2.5, d’(NI)=S/**N**_**o**_ in Eq 1, with α_NI_=**N**_**o**_=1.0. Eq. 2 was best-fit to the paired d’(IM) scores with α_IM_=2.1, β=2.0 (dotted line), although the fit is loose (root-mean square error, RMSE, being 0.17). The RGL data ratios σ(IM)/σ(NI) are plotted in Fig. 8 (top right) by black circles along with Eq. 3 (β=2.0, k best fit to 2.5) (dotted curve) (r=+0.97).
(14)  Page 18, lines49-50. Eq 1 best-fit with α_NI_=1.0. Eq 2 was fit with α_IM_=2.2; β=4.2. (RMSE = 0.09). Middle right: ratios σ(IM)/σ(NI) from the data. The dotted line plots Eq 3 with k best fit to 1.10.
(15)  Page 18, lines 52-55. Eq 1 was best-fit with α_NI_=1.0. Eq 2 best-fit with α_IM_=4.0, β=5.0; the reverse Perky at low d’(NI) being clear-cut (RMSE=0.11). Bottom right: ratios σ(IM)/σ(NI) from the data (black circles). The dotted line plots Eq. 3 with k=1.15.
(16)  Page 22, lines 24-27.

This can be explained in terms of Eq. 2 if the external noise from the masking pattern overwhelmed the multiplicative noise term, βS. To test this, best-fits to d’(IM) with Eq 2 were obtained with α_NI_=1, σ_IMp_=1.05, σ_IMo_=0.88, and β=0.05 in IMp and 0.02 in IMo (RMSE=0.11). Thus βS<<N_**o**_=1 at every level of S, as hypothesized.

(17)  Page 22, line 29: Thus the ratio σ(IM)/σ(NI) should not noticeably increase..
(18)  Page 23, lines 18-19. These results can be explained in the context of Eq 2 if images boost similar (‘congruent’) stimuli (α_NI_>1) but not dissimilar ones (α_NI_ =1).
(19)  Page 26, lines 26-27. Similar performance in NI and IM implies that S is the same, so their memory effect must be carried by α_IM_ or β in Eq.2; for example, α_IM_ might be larger in the STM than in the LTM condition due to relative recency.

